# Trends, predictors, and association of surgical timing with mortality among patients with *C*lostridioides difficile infection requiring colectomy: a cohort study

**DOI:** 10.1017/ice.2026.10411

**Published:** 2026-05

**Authors:** Zhengxi Chen, Xiuhan Li, Jesse Fajnzylber, William M Patterson, James Townley, Abhishek Deshpande

**Affiliations:** 1 Case Western Reserve University, Cleveland, USA; 2 Johns Hopkins University, USA; 3 Mayo Clinic in Rochester, USA; 4 Johns Hopkins Medicine, USA; 5 Alice L Walton School of Medicine, USA

## Abstract

**Background:**

*Clostridioides difficile* infection (CDI) requiring colectomy carries substantial mortality risk, with optimal timing of surgery remaining poorly defined. We examined temporal trends in colectomy among inpatients with CDI, identified predictors of surgical intervention and postoperative mortality, and evaluated the association between surgical timing and patient outcomes.

**Methods:**

A retrospective cohort study was conducted using the National Inpatient Sample database from 2018 to 2022. We compared patients undergoing colectomy with those managed medically. To minimize confounding by hospital-onset cases, the analysis of surgical timing and mortality was restricted to patients undergoing colectomy within 8 days of admission. Predictors were identified using survey-weighted logistic regression and LASSO regression models.

**Results:**

Among 240,564 CDI hospitalizations (representing 1,207,995 weighted nationally), 717 patients underwent colectomy (3,585 weighted). CDI prevalence declined from 0.99% (2018) to 0.76% (2022), while colectomy rates increased from 0.28% to 0.34%. Peritonitis (OR 5.42; 95% CI, 4.46–6.59), coagulopathy (OR 4.96; 95% CI, 3.76–6.55), and sepsis/septic shock (OR 3.89; 95% CI, 3.39–4.47) were the strongest predictors of colectomy. Among patients undergoing colectomy within 8 days (2,830 weighted), in-hospital mortality was 26.5% overall, increasing from 21.0% (2018) to 30.7% (2022). Sepsis/septic shock (OR 8.20; 95% CI 2.92–23.07) and coagulopathy (OR 7.27; 95% CI 3.31–15.97) predicted mortality. Each additional day from admission to colectomy was associated with a 16% (OR 1.16; 95% CI 1.04–1.28) increased mortality risk.

**Conclusions:**

In this nationally representative cohort, surgical timing was an independent and modifiable determinant of survival in patients with CDI requiring colectomy. Our findings underscore the importance of early surgical consultation for CDI patients with peritonitis, sepsis, and coagulopathy.

## Introduction

*Clostridioides difficile* infection (CDI) is one of the most common causes of healthcare-associated diarrhea in the United States, accounting for >500,000 cases and 30,000 deaths annually.^1^ Most patients are treated with oral antibiotic therapy, with vancomycin or fidaxomicin being the first-line treatments.^2,3^ However, not all patients respond to antibiotic therapy, and treatment can be challenging in those who develop fulminant CDI. Approximately, 1%–3% of patients develop fulminant CDI, characterized by rapid progression to extensive colonic inflammation and systemic toxicity.^4,5^

Approximately 1% of all patients with CDI and 30% with fulminant CDI require emergency surgery.^2^ The subset of patients requiring surgical intervention presents significant management challenges with mortality rates ranging from 20%–85%.^6^ Evidence suggests that delayed surgical intervention may compromise patient outcomes, with increased mortality rates observed even after operative management.^7,8^ Therefore, timing of surgical intervention represents an important determinant of survival in patients with severe/fulminant CDI.

Emergency colectomy for CDI is typically reserved for patients with fulminant colitis, colonic perforation, toxic megacolon, or those failing to respond to maximal medical therapy. The decision to proceed with surgery involves consideration of multiple factors, including patient comorbidities, CDI severity, and timing of intervention. Previous studies have identified various predictors of surgical need and postoperative outcomes, including development of sepsis, preoperative shock, coagulopathy, advanced age, and immunosuppression.^2,9,10^ However, these studies are often limited by small sample sizes, single-center designs, or analysis of data from earlier treatment eras.

While recent clinical guidelines emphasize early surgical consultation and multidisciplinary decision-making, there are limited data on the real-world implementation of these recommendations and their impact on patient outcomes.^3,11^ Using a national inpatient dataset, we conducted a retrospective cohort study of inpatients with CDI requiring colectomy from 2018 to 2022 to examine trends, identify predictors of surgical colectomy and mortality, and evaluate the impact of surgical timing on patient outcomes.

## Methods

### Data source

We used the National Inpatient Sample (NIS), developed by the Agency for Healthcare Research and Quality (AHRQ) and maintained by the Healthcare Cost and Utilization Project (HCUP) for the years 2018–2022. A detailed description of the NIS database is provided as Supplementary material 1A. Approval for the use of NIS data for this study was obtained from the institutional review board of Cleveland Clinic and HCUP. All analytical procedures adhered to the methodological standards for NIS data analysis as recommended by AHRQ.^12^

### Study population

**Fig. 1.**
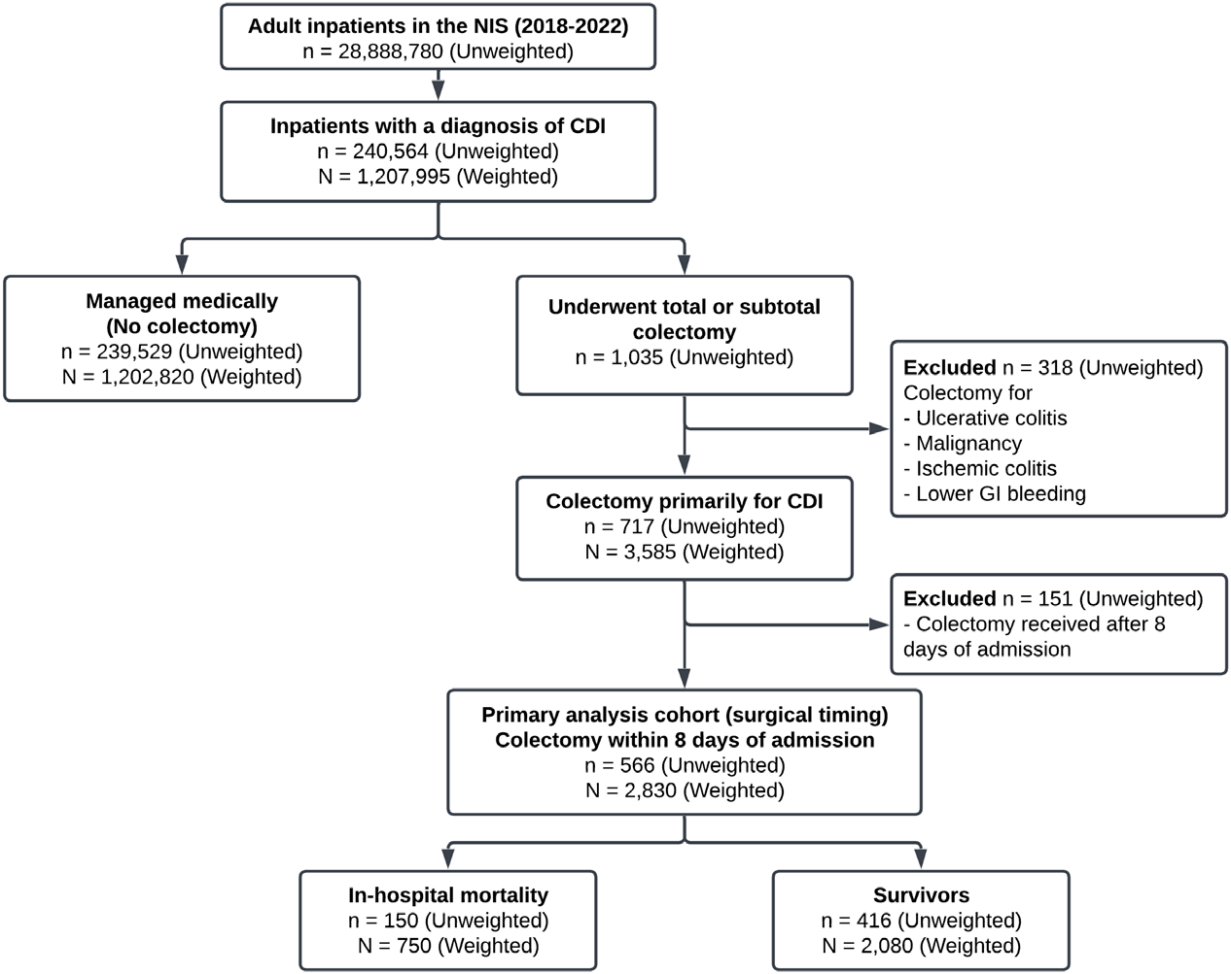
Consolidated standards of reporting (CONSORT) Figure describing the flow of patient discharges based on the inclusion/exclusion criteria.

We identified adult patients (≥18 years) hospitalized between 2018 and 2022 with primary or secondary diagnoses of CDI using ICD-10-CM codes A04.71 and A04.72. Among patients with CDI, we identified those who underwent total or subtotal colectomy using specific ICD-10-CM procedure codes (Table S1). To ensure colectomy was performed primarily for CDI complications, we excluded patients with concurrent diagnoses that could independently necessitate colectomy: ulcerative colitis, malignancy, ischemic colitis, and lower gastrointestinal bleeding. To minimize confounding by hospital-onset cases, the analysis of surgical timing was restricted to patients undergoing colectomy within 8 days of admission. We excluded patients with missing data for key variables: age, sex, primary payer, hospital characteristics, length of stay, and discharge disposition. Figure 1 outlines the systematic implementation of inclusion and exclusion criteria for patient selection and cohort establishment for comparison. All study patients were followed until discharge from or death in the hospital.

The relationships between colectomy and outcomes were analyzed at two levels. First, to identify risk factors for surgical intervention among patients with CDI, we compared patients who underwent colectomy with those managed medically without surgery. Second, among the subset of patients who underwent colectomy within 8 days of admission, we compared survivors with non-survivors to identify predictors of postoperative mortality and evaluate the impact of surgical timing on outcomes.

### Outcomes and covariates determination

The primary outcomes of interest were (1) prevalence of CDI among all hospitalizations, (2) prevalence of colectomy among patients with CDI, and (3) in-hospital mortality following colectomy for CDI. Patients discharged alive were considered survivors for mortality analyses, as we did not have access to postdischarge vital status or readmissions. Secondary outcomes included emergency department (ED) admission rates, hospital length of stay (LOS), postoperative complications (cerebrovascular accident, pulmonary complications, cardiac complications, acute renal failure, postoperative bleeding, and urinary tract infection), total hospitalization charges, and discharge disposition.^13^ Covariates included patient demographics, in-hospital characteristics, CDI-related complications (electrolyte abnormalities, sepsis/septic shock, coagulopathy, peritonitis, and colonic perforation), and comorbidity profile captured by the Elixhauser Comorbidity Software Refined for ICD-10-CM Diagnosis, v2025.1.^14,15^ Detailed definitions are provided in Supplementary material 1B, Table S1 and Table S2.

### Statistical analysis

We used NIS survey weights for all analyses to generate nationally representative estimates. The complex survey design was accounted for by designating hospital identification numbers as primary sampling units with strata defined by the interaction of NIS stratum and year.

We calculated the annual weighted frequency and prevalence of CDI, colectomy among patients with CDI, and mortality among colectomy patients with CDI with 95% confidence intervals. To determine if these trends were statistically significant, we performed a weighted logistic regression controlling for age, sex, and race. We also compared patient demographics, clinical characteristics, comorbidities, complications, and outcomes between patients who underwent colectomy versus those who did not, and between survivors and non-survivors following colectomy. Categorical variables were summarized as weighted percentages and compared using weighted Pearson’s chi-square tests with the Rao-Scott adjustment. Continuous variables were summarized as medians with interquartile ranges and compared using Mann-Whitney U tests. Bonferroni correction was applied to all pairwise comparisons to control familywise error rate.

To identify factors associated with (a) colectomy among patients with CDI and (b) mortality among colectomy patients, we employed two multivariable modeling approaches. First, survey-weighted logistic regression with stepwise variable selection based on the Akaike Information Criterion (AIC) was performed. Second, least absolute shrinkage and selection operator (LASSO) regression with 10-fold cross-validation was used for simultaneous variable selection and regularization, with the optimal lambda parameter selected using the one-standard-error rule. Model discrimination was assessed using the area under the receiver operating characteristic curve (AUC), with 95% confidence intervals calculated using bootstrap methods. ROC curves were generated for both models to visualize performance, and the DeLong test compared AUC values between the two modeling approaches. Statistical significance was set at *P* < .05 (two-sided). Analyses were performed using SAS version 9.4 (SAS Institute, Cary, NC) and R Studio (R Core Team (2023).

## Results

**Fig. 2.**
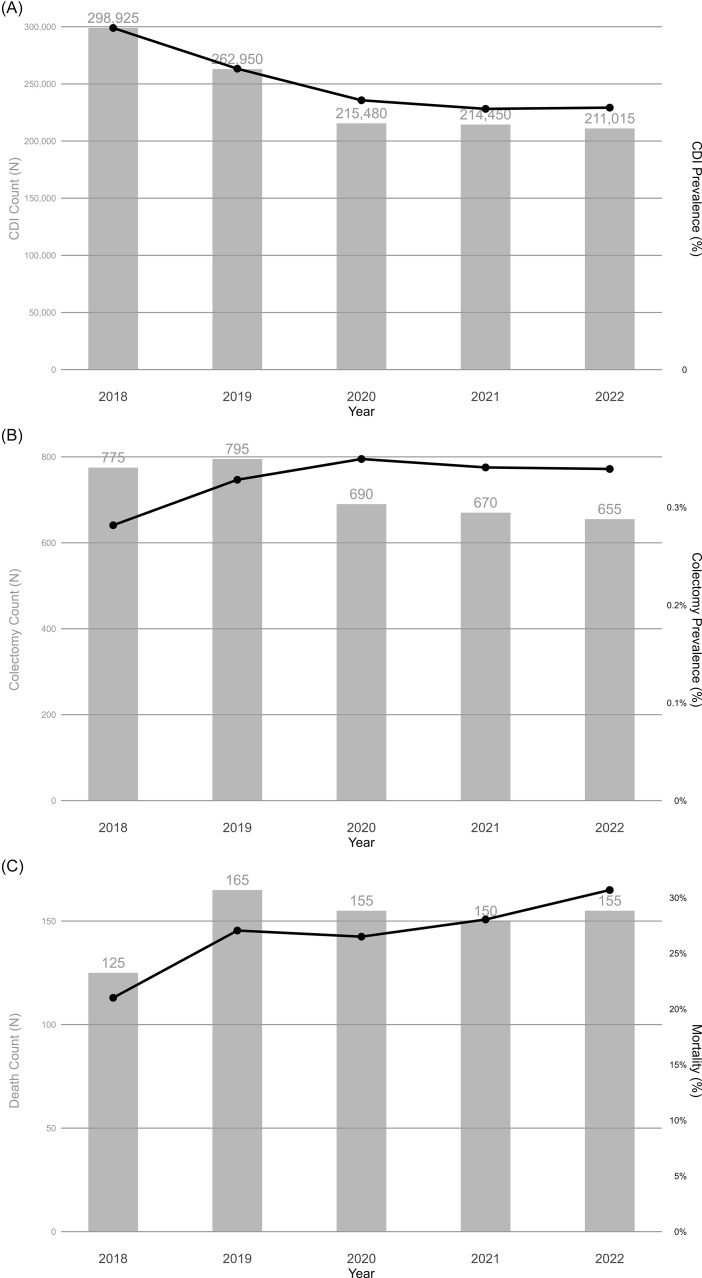
Annual number and prevalence of (A) *Clostridioides difficile* infection, (B) colectomy among patients with *Clostridioides difficile* infection, (C) death among patients with *Clostridioides difficile* after colectomy within 8 days of admission from 2018 to 2022.

We identified 240,564 inpatients with CDI diagnoses, representing 1,207,995 inpatients nationally after applying NIS survey weights. As shown in Table 1, CDI prevalence declined from 0.99% (95% CI: 0.91%–1.01%) in 2018 to 0.76% (95% CI: 0.74%–0.78%) in 2022 (Fig. 2A). Among patients with CDI, prevalence of colectomy increased from 0.28% (95% CI: 0.24%–0.33%) in 2018 to 0.34% (95% CI: 0.28%–0.40%) in 2022 (Fig. 2B). In-hospital mortality among patients who underwent colectomy within 8 days of admission increased from 21.01% (95% CI: 13.87%–28.15%) in 2018 to 30.69% (95% CI: 21.75%–39.63%) in 2022 (Fig. 2C). However, these observed temporal trends were not statistically significant (*P* > .05).

**Table 1.** Prevalence of *Clostridioides difficile* infection, colectomy rates, and postoperative mortality in the United States, 2018–2022

Outcome	2018	2019	2020	2021	2022
**CDI**					
N (95% CI)	298,925 (284,609, 313,241)	262,950 (250,371, 275,529)	215,480 (205,108, 225,852)	214,450 (203,850, 225,050)	211,015 (200,543, 221,487)
Prevalence (%) (95% CI)	0.99 (0.91, 1.01)	0.87 (0.85, 0.89)	0.78 (0.76, 0.80)	0.75 (0.73, 0.77)	0.76 (0.74, 0.78)
**Colectomy for CDI**					
N (95% CI)	775 (715, 835)	795 (739, 851)	690 (640, 740)	670 (609, 731)	655 (598, 712)
Prevalence (%) (95% CI)	0.28 (0.24, 0.33)	0.33 (0.28, 0.38)	0.35 (0.29, 0.41)	0.34 (0.28, 0.40)	0.34 (0.28, 0.40)
**Colectomy for CDI within 8 days of admission**					
N (95% CI)	595 (539, 651)	610 (555, 665)	585 (535, 635)	535 (483, 587)	505 (449, 561)
**Mortality following colectomy within 8 days of admission**					
Deaths (N) (95% CI)	125 (82, 168)	165 (111, 219)	155 (104, 206)	150 (108, 192)	155 (110, 200)
Mortality (%) (95% CI)	21.01 (13.87, 28.15)	27.05 (18.79, 35.31)	26.50 (17.92, 35.07)	28.04 (19.97, 36.10)	30.69 (21.75, 39.63)

CDI = *Clostridioides difficile* infection. Note. N = weighted count, CI = confidence interval.

### Predictors of colectomy among patients with CDI

Among 240,564 (representing 1,207,995 nationally) inpatients with CDI, 717 (0.30%, representing 3,585 nationally) underwent colectomy. Table 2 compares characteristics between CDI patients underwent colectomy and those without. Detailed description is provided in Supplementary material 1C. Despite lower overall comorbidity burden, patients who underwent colectomy had substantially higher rates of severe CDI complications: electrolyte abnormalities (80.3% vs 60.2%), sepsis/septic shock (72.8% vs 33.0%), coagulopathy (7.0% vs 0.6%), peritonitis (23.5% vs 2.2%), and colonic perforation (10.8% vs 0.5%) (all *P* < .001). Patients who underwent colectomy experienced worse clinical outcomes, including higher mortality rates (25.3% vs 6.4%), lower home discharge rate (11.4% vs 38.8%), longer median hospital LOS (16 vs 6 days), and higher in-hospital costs ($241,976 vs $56,322) (all *P* < .001).

**Table 2.** Baseline characteristics, comorbidities, complications, and outcomes of patients hospitalized with *Clostridioides difficile* infection, stratified by colectomy status

Factor	Level	Colectomy (N = 5,175)	No-colectomy (N = 1,202,820)	*P*-Value	*P*-correction
**Demographics**					
Age (yr), median [Q1, Q3]	–	66 [56,75]	69 [57,79]	<.001*	<.001*
Sex, %	Female	54.6	56.9	.14	>.9
	Male	45.4	43.1		
Race, %	White	75.9	73.3	.04	>.9
	Black	10.2	13.1		
	Hispanic	9.3	8.6		
	Other	4.6	5.0		
Primary payer, %	Medicare	56.9	65.1	<.001*	<.001*
	Medicaid	13.4	12.4		
	Private	24.0	17.9		
	Other	5.8	4.6		
Median household income quartiles by zip code, %	0–25th Percentile	28.2	28.2	.55	>.9
	26th to 50th Percentile	27.8	26.8		
	51st to 75th Percentile	25.3	24.6		
	76th to 100th Percentile	18.7	20.5		
Location, %	“Central” counties of metro areas of >= 1 million population	30.2	27.4	.09	>.9
	“Fringe” counties of metro areas of >= 1 million population	23.8	25.0		
	Counties in metro areas of 250,000–999,999 population	19.2	20.9		
	Counties in metro areas of 50,000–249,999 population	9.4	10.2		
	Micropolitan counties	11.3	9.5		
	Not metropolitan or micropolitan counties	6.1	7.0		
Discharge year, %	2018	23.1	24.9	.57	>.9
	2019	21.8	21.9		
	2020	19.7	17.9		
	2021	17.7	17.8		
	2022	17.7	17.5		
Discharge quarter, %	1st	27.5	26.0	.63	>.9
	2nd	23.4	24.9		
	3rd	24.7	24.8		
	4th	24.4	24.4		
**In-hospital characteristics**					
Admission day, %	Weekday	77.7	75.9	.19	>.9
	Weekend	22.3	24.1		
Admission type, %	Elective	9.6	6.6	<.001*	.007
	Non-elective	90.4	93.4		
Hospital: census division, %	New England	4.7	5.2	.44	>.9
	Middle Atlantic	13.9	13.5		
	East North Central	18.7	17.5		
	West North Central	6.6	7.5		
	South Atlantic	19.9	20.3		
	East South Central	7.2	6.6		
	West South Central	8.7	10.0		
	Mountain	8.4	7.0		
	Pacific	11.9	12.4		
Hospital: location/teaching status, %	Rural	5.6	9.0	<.001*	<.001*
	Urban nonteaching	12.8	18.4		
	Urban teaching	81.6	72.5		
Hospital: region, %	Northeast	18.7	18.7	.84	>.9
	Midwest	25.3	25.0		
	South	35.8	36.9		
	West	20.3	19.3		
Hospital: control/ownership, %	Government nonfederal	13.5	11.3	.002	.1
	Private not-profit	78.8	78.2		
	Private invest-own	7.6	10.5		
Hospital: bedsize, %	Small	15.1	21.6	<.001*	<.001*
	Medium	25.3	27.6		
	Large	59.6	50.8		
Transfer in indicator, %	Transferred in from a different acute care hospital	15.3	8.5	<.001*	<.001*
	Transferred in from another type of health facility	6.8	7.4		
	Not a transfer	77.9	84.1		
Transfer out indicator, %	Transferred out to a different acute care hospital	1.7	2.3	<.001*	<.001*
	Transferred out to another type of health facility	41.4	30.9		
	Not a transfer	57.0	66.8		
**Comorbidity profile**					
AIDS, %	Yes	.4	.8	.11	>.9
Alcohol abuse, %	Yes	4.1	7.2	<.001*	.004*
Autoimmune conditions, %	Yes	5.2	5.6	.55	>.9
Lymphoma,, %	Yes	1.8	2.6	.14	>.9
Leukemia, %	Yes	1.8	2.0	.70	>.9
Metastatic cancer, %	Yes	4.5	5.5	.21	>.9
Solid tumor without metastasis in situ, %	Yes	.0	.1	.50	>.9
Solid tumor without metastasis, malignant, %	Yes	4.7	5.4	.33	>.9
Dementia, %	Yes	5.4	10.5	<.001*	<.001*
Depression, %	Yes	15.6	17.0	.21	>.9
Diabetes without chronic complications, %	Yes	4.9	7.8	<.001*	.02*
Diabetes with chronic complications, %	Yes	21.4	26.7	<.001*	.005*
Drug abuse, %	Yes	2.6	3.8	.048	>.9
Hypertension, complicated, %	Yes	31.9	37.8	<.001*	.004*
Hypertension, uncomplicated, %	Yes	22.2	30.3	<.001*	<.001*
Chronic pulmonary disease, %	Yes	21.2	23.8	.05	>.9
Obesity, %	Yes	17.5	15.9	.16	>.9
Peripheral vascular disease, %	Yes	8.0	8.2	.80	>.9
Hypothyroidism, %	Yes	14.2	17.2	.01	.40
Other thyroid disorders, %	Yes	1.0	1.3	.40	>.9
Elixhauser comorbidity index for risk of 30-day, all-cause readmission, median [Q1, Q3]	–	2(0,6)	4(0,7)	<.001*	<.001*
Elixhauser comorbidity index for risk of in-hospital mortality, median [Q1, Q3]	–	0 (−5, 2)	0 (−5, 1)	.70	>.9
**Complications**					
Electrolyte abnormalities, %	Yes	80.3	60.2	<.001*	<.001*
Sepsis/septic shock, %	Yes	72.8	33.0	<.001*	<.001*
Coagulopathy, %	Yes	7.0	.6	<.001*	<.001*
Peritonitis, %	Yes	23.5	2.2	<.001*	<.001*
Colonic perforation, %	Yes	10.8	.5	<.001*	<.001*
**Health outcomes**					
LOS (day), median [Q1, Q3]	–	16 (9,27)	6 (4,12)	<.001*	–
Mortality, %	Yes	25.3	6.4	<.001*	–
ED, %	Yes	71.2	80.7	<.001*	–
Home discharge, %	Yes	11.4	38.8	<.001*	–
Cost, median [Q1, Q3]	–	241,976 (140,900,417,990)	56,322 (29,398,121,897)	<.001*	–

In multivariable survey-weighted logistic regression with stepwise selection (Table 3), CDI-related complications were the strongest predictors of colectomy. Peritonitis conferred the highest odds (OR 5.42, 95% CI 4.46–6.59), followed by colonic perforation (OR 5.18, 95% CI 3.90–6.90), coagulopathy (OR 4.96, 95% CI 3.76–6.55), and sepsis/septic shock (OR 3.89, 95% CI 3.39–4.47). Hospital factors associated with increased risk included elective admission (OR 2.00, 95% CI 1.60–2.50), transfers in from other acute care hospitals (OR 1.35, 95% CI 1.12–1.63), transfers out to other health facilities (OR 1.55, 95% CI 1.36–1.77), urban teaching status (OR 1.38, 95% CI 1.03–1.84) and large hospitals (OR 1.36, 95% CI 1.13–1.63). Several comorbidities were associated with lower odds of colectomy, including alcohol abuse (OR 0.44, 95% CI 0.32–0.60), uncomplicated diabetes (OR 0.69, 95% CI 0.52–0.93), dementia (OR 0.59, 95% CI 0.44–0.78) and uncomplicated hypertension (OR 0.74, 95% CI 0.62–0.87).

**Table 3.** Predictors of colectomy and mortality following colectomy using survey-weighted stepwise logistic regression and least absolute shrinkage and selection operator (LASSO) regression

		Distribution	Stepwise logistic regression		LASSO regression
**Variable**	**Level**	**No. (%)** ^a^	**aOR (95% CI)**	***P*-Value**	**Odds ratio** ^b^
**A. Predictors of colectomy**		1,207,995			
**Demographics**					
Age	Continuous	—	0.995 (0.990, 0.999)	.048*	1.00
Race	White	885,595 (73.3%)	—	—	1.09
Primary care	Private	216,547(17.9%)	1.34 (1.07, 1.67)	.01*	1.10
	Other	55,630 (4.6%)	1.39 (1.01, 1.90)	.04*	—
**CDI complications**					
Peritonitis	Yes	27,678 (2.3%)	5.42 (4.46, 6.59)	<.001*	5.87
Colonic perforation^c^	Yes	60,700 (5.0%)	5.18 (3.90, 6.90)	<.001*	5.28
Coagulopathy	Yes	7,579 (0.6%)	4.96 (3.76, 6.55)	<.001*	4.81
Sepsis/septic shock	Yes	400,698 (33.2%)	3.89 (3.39, 4.47)	<.001*	3.29
Electrolyte abnormalities	Yes	728,253 (60.3%)	1.89 (1.63, 2.20)	<.001*	1.51
**Hospital factors**					
Admission type	Elective	79,883 (6.6%)	2.00 (1.60, 2.50)	<.001*	1.48
Transfer in	Acute Care Hosp.	103,031 (8.5%)	1.35 (1.12, 1.63)	.002*	1.28
Transfer out	Other facility	373,814 (30.9%)	1.55 (1.36, 1.77)	<.001*	1.22
Teaching status	Urban teaching	876,267 (72.5%)	1.38 (1.03, 1.84)	.03*	1.18
Bed size	Large	614,117 (50.8%)	1.36 (1.13, 1.63)	<.001*	1.08
Control/ownership	Private invest-own	126,689 (10.5%)	0.64 (0.48, 0.85)	.002*	0.93
**Comorbidities**					
Alcohol abuse	Yes	86,815 (7.2%)	0.44 (0.32, 0.60)	<.001*	0.80
Dementia	Yes	126,576 (10.5%)	0.59 (0.44, 0.78)	<.001*	0.85
Diabetes without chronic complications	Yes	94,074 (7.8%)	0.69 (0.52, 0.93)	.01*	—
Diabetes with chronic complications	Yes	322,260 (26.7%)	0.79 (0.67, 0.93)	.005*	—
Hypertension uncomplicated	Yes	365,603 (30.3%)	0.74 (0.62, 0.87)	<.001*	0.97
Hypertension complicated	Yes	456,317 (37.8%)	0.70 (0.59, 0.82)	<.001*	0.92
Elixhauser comorbidity index for risk of 30-day, all-cause readmission	Continuous	—	—	—	0.97
**B. Predictors of mortality**		2,830			
**Surgical timing**	Continuous	—	1.16 (1.04,1.28)	.01*	1.01
**Demographics**					
Age	Continuous	—	1.03 (1.01,1.05)	.001*	1.01
**CDI complications**					
Sepsis/septic shock	Yes	2,275 (80.4%)	8.20 (2.92,23.07)	<.001*	2.36
Coagulopathy	Yes	244 (8.6%)	7.27 (3.31,15.97)	<.001*	2.80
**Hospital factors**					
Admission Type	Elective	297 (10.5%)	0.26 (0.08,0.82)	.02*	—
**Comorbidities**					
Alcohol abuse	Yes	81 (2.9%)	5.73 (1.68,19.57)	.01*	1.17
Elixhauser comorbidity index for risk of in-hospital mortality	Continuous	—	1.05 (1.01,1.09)	.02*	1.01

aOR, adjusted odds ratio; CI, confidence interval; CDI, *Clostridioides difficile* infection; LASSO, Least Absolute Shrinkage and Selection Operator.

a Represents the weighted number and percentage of patients in the respective cohort (Total CDI cohort for Section A; Colectomy cohort within 8 days after admission for Section B).

b LASSO regression coefficients were converted to odds ratios. Variables with coefficient shrunk to zero are indicated by “—”.

c Referred to as “Colon disorders” in the previous Stepwise model; harmonized to “Colonic perforation” for clarity.

As shown in Table 3, the LASSO regression model identified similar predictors, with peritonitis (OR 5.87), colonic perforation (OR 5.28), coagulopathy (OR 4.81), and sepsis/septic shock (OR 3.29) as the strongest predictors. Hospital factors associated with increased odds of colectomy included elective admissions (OR 1.48), transfers from other acute care hospitals (OR 1.28), transfers out from other facilities (OR 1.22), urban teaching hospitals (OR 1.18) and large hospitals (OR 1.08). Both modeling approaches demonstrated good discrimination for predicting colectomy, with AUC values of 0.82 (95% CI: 0.81–0.84) for stepwise logistic regression and 0.82 (95% CI: 0.80–0.83) for LASSO regression (Fig. S1). The two models’ discriminative abilities were not significantly different by DeLong’s test (*P* = .38), suggesting comparable predictive performance despite different variable selection approaches.

### Predictors of mortality among patients who underwent colectomy

Among patients who underwent colectomy (unweighted N = 717; weighted N = 3,585), 2,830 (78.94%) received colectomy within 8 days of admission, with 1,810 (50.48%) undergoing colectomy within 0–2 days. For patients who received colectomy within 8 days of admission, the weighted mortality during hospitalization was 26.50% (95% CI: 22.82%–30.19%). Table 4 presents comparisons between survivors and non-survivors. After Bonferroni correction for multiple comparisons, several factors differed significantly between groups. Non-survivors had higher rates of severe complications including sepsis/septic shock (97.3% vs 74.3%, *P* < .001), and coagulopathy (22.0% vs 3.8%, *P* < .001). Survivors were more likely to have elective admissions (13.3% vs 2.7%, *P* = .01). For postoperative complications, survivors had significantly lower risk of cardiac complications (6.0% vs 12.0%, *P* = .02) and postoperative bleeding (0.0% vs 2.0%, *P* = .004), but higher risk of urinary tract infection (20.2% vs 14.0%, *P* = .01).

**Table 4.** Baseline characteristics, comorbidities, complications, and outcomes of patients undergoing colectomy within eight days of admission for *Clostridioides difficile* infection, stratified by survival status

Factor	Level	Non-survivors (N = 750)	Survivors (N = 2,080)	*P*-Value	*P* correction
**Demographics**					
Age (yr), median [Q1, Q3]	–	71 (63, 77)	67 (57, 76)	.01*	>.3
Sex, %	Female	62.0	61.1	.84	>.9
	Male	38.0	38.9		
Race, %	White	75.0	76.9	.79	>.9
	Black	9.5	10.7		
	Hispanic	10.1	8.5		
	Other	5.4	4.0		
Primary payer, %	Medicare	71.8	61.8	.16	>.9
	Medicaid	10.1	11.8		
	Private	14.1	19.5		
	Other	4.0	7.0		
Median household income quartiles by zip code, %	0–25th Percentile	34.2	27.9	.41	>.9
	26th to 50th Percentile	28.8	27.7		
	51st to 75th Percentile	24.0	28.2		
	76th to 100th Percentile	13.0	16.3		
Location, %	“Central” counties of metro areas of >= 1 million population	25.0	31.0	.47	>.9
	“Fringe” counties of metro areas of >= 1 million population	23.0	25.4		
	Counties in metro areas of 250,000–999,999 population	18.9	18.4		
	Counties in metro areas of 50,000–249,999 population	10.8	9.2		
	Micropolitan counties	13.5	10.2		
	Not metropolitan or micropolitan counties	8.8	5.8		
Discharge year, %	2018	16.7	22.6	.58	>.9
	2019	22.0	21.4		
	2020	20.7	20.7		
	2021	20.0	18.5		
	2022	20.7	16.8		
Discharge quarter, %	1st	24.8	27.5	.70	>.9
	2nd	24.1	23.3		
	3rd	24.8	27.3		
	4th	26.2	21.9		
**In-hospital characteristics**					
Admission day, %	Weekday	78.7	76.2	.54	>.9
	Weekend	21.3	23.8		
Admission type, %	Elective	2.7	13.3	<.001*	.01*
	Non-elective	97.3	86.7		
Hospital: census division, %	New England	3.3	6.2	.64	>.9
	Middle Atlantic	12.7	13.7		
	East North Central	14.7	18.3		
	West North Central	6.0	6.7		
	South Atlantic	21.3	21.2		
	East South Central	10.0	6.5		
	West South Central	12.0	8.7		
	Mountain	8.0	8.7		
	Pacific	12.0	10.1		
Hospital: location/teaching status, %	Rural	8.0	5.3	.28	>.9
	Urban Nonteaching	17.3	14.2		
	Urban Teaching	74.7	80.5		
Hospital: region, %	Northeast	16.0	20.0	.35	>.9
	Midwest	20.7	25.0		
	South	43.3	36.3		
	West	20.0	18.7		
Hospital: control/ownership, %	Government Nonfederal	15.3	11.8	.37	>.9
	Private Not-profit	78.0	78.8		
	Private Invest-own	6.7	9.4		
Hospital: bedsize, %	Small	16.7	15.9	>.9	>.9
	Medium	24.7	26.2		
	Large	58.7	57.9		
Transfer in indicator, %	Transferred in from a different acute care hospital	18.1	13.3	.28	>.9
	Transferred in from another type of health facility	10.1	8.7		
	Not A Transfer	71.8	78.0		
Transfer out indicator, %	Transferred out to a different acute care hospital	0.0	2.4	–	–
	Transferred out to another type of health facility	0.0	61.8		
	Not a Transfer	100.0	35.8		
**Comorbidity profile**					
AIDS, %	Yes	0.0	0.7	.30	>.9
Alcohol abuse, %	Yes	4.7	2.2	.11	>.9
Autoimmune conditions, %	Yes	2.7	7.7	.03*	>.9
Lymphoma, %	Yes	3.3	2.2	.43	>.9
Leukemia, %	Yes	6.0	1.4	.003*	.15
Metastatic cancer, %	Yes	6.0	4.1	.35	>.9
Solid tumor without metastasis in situ, %	Yes	0.0	0.0	–	–
Solid tumor without metastasis, malignant, %	Yes	3.3	3.1	.90	>.9
Dementia, %	Yes	6.0	5.8	>.9	>.9
Depression, %	Yes	10.0	17.1	.04*	>.9
Diabetes without chronic complications, %	Yes	5.3	4.6	.71	>.9
Diabetes with chronic complications, %	Yes	21.3	23.8	.53	>.9
Drug abuse, %	Yes	0.0	3.4	.02*	>.9
Hypertension, complicated, %	Yes	38.0	33.9	.35	>.9
Hypertension, uncomplicated, %	Yes	20.7	26.2	.18	>.9
Chronic pulmonary disease, %	Yes	30.7	21.2	.02*	.65
Obesity, %	Yes	18.0	18.7	.84	>.9
Peripheral vascular disease, %	Yes	9.3	7.7	.53	>.9
Hypothyroidism, %	Yes	15.3	17.1	.62	>.9
Other thyroid disorders, %	Yes	0.0	1.0	.22	>.9
Elixhauser comorbidity index for risk of 30-day, all-cause readmission, median [Q1, Q3]	–	4 (0, 7)	2 (0, 6)	.15	>.9
Elixhauser comorbidity index for risk of in-hospital mortality, median [Q1, Q3]	–	0 (-3, 3)	0 (−7, 1)	<.001*	.02*
**CDI complications**					
Electrolyte abnormalities, %	Yes	91.3	82.0	.01*	.31
Sepsis/septic shock, %	Yes	97.3	74.3	<.001*	<.001*
Coagulopathy, %	Yes	22.0	3.8	<.001*	<.001*
Peritonitis, %	Yes	24.7	23.8	.83	>.9
Colonic perforation, %	Yes	10.7	7.7	.26	>.9
Colectomy day, median [Q1, Q3]	–	2 (0, 4)	2 (1, 4)	.004*	.19
**Health outcomes**					
LOS (day), median [Q1, Q3]	–	7 (3, 13)	16 (11, 23)	<.001*	–
ED, %	Yes	78.7	71.2	.08	–
Cost, median [Q1, Q3]	–	196,553 (115,414, 287,829)	232,546 (125,881, 362,563)	.03*	–
**Postoperative complications**					
Cerebrovascular accident	Yes	0.0	0.0	–	–
Pulmonary complications, %	Yes	1.3	0.2	.12	
Cardiac complications, %	Yes	12.0	6.0	.02*	–
Acute renal failure, %	Yes	44.0	40.6	.50	–
Postoperative bleeding, %	Yes	2.0	0.0	.004*	–
Urinary tract infection, %	Yes	14.0	20.2	.01*	–

In multivariable regression analysis, important predictors of mortality included sepsis/septic shock (OR 8.20, 95% CI 2.92–23.07), coagulopathy (OR 7.27, 95% CI 3.31–15.97), alcohol abuse (OR 5.73, 95% CI 1.68–19.57), higher mortality risk score (OR 1.05, 95% CI 1.01–1.09) and increasing age (OR 1.03 per year, 95% CI 1.01–1.05) (Table 3). Surgical timing remained independently associated with mortality risk. Each additional day from admission to colectomy was associated with a 16% (OR 1.16, 95% CI 1.04–1.28) increased mortality risk. The LASSO regression model identified similar predictors for mortality, including delayed surgery (OR = 1.01), sepsis/septic shock (OR = 2.25), coagulopathy (OR = 2.61), alcohol abuse (OR = 1.17), increasing age (OR = 1.01), and higher mortality risk score (OR = 1.01) (Table 3). Discrimination for predicting mortality did not differ significantly between the stepwise multivariable logistic regression model (AUC 0.78, 95% CI 0.74–0.82) and the LASSO model (AUC 0.76, 95% CI 0.71–0.80; DeLong’s test *P* = .49) (Fig. S2).

## Discussion

In this large, nationally representative retrospective cohort study of >240,000 inpatients with CDI across the United States, we found that the overall prevalence of CDI has declined, and about 0.3% of patients required colectomy for severe complications. Colectomy for CDI was associated with significant mortality risk, with nearly one in three patients experiencing mortality during hospitalization. The key predictors for colectomy were CDI-related complications including peritonitis and sepsis, while predictors for mortality after colectomy included sepsis and coagulopathy. Most importantly, our data demonstrates that surgical timing significantly impacted outcomes, with early colectomy associated with improved survival compared to delayed intervention. Our findings suggest that physicians may optimize outcomes through early recognition of surgical indications. However, overall mortality rates remained high, highlighting the severity of CDI cases requiring operative management.

Our findings are consistent with recent epidemiological studies showing decreasing trends in CDI prevalence in the US over recent years. According to the Centers for Disease Control and Prevention (CDC), the national CDI burden in the United States showed a 4% annual decrease from 2011 to 2017.^16^ This could be attributed to improved diagnostic strategies, newer treatments, antimicrobial stewardship, and infection control initiatives across healthcare systems.

Several other studies have reported continued CDI reduction from 2017 to 2021, especially during the COVID-19 pandemic.^17–19^ The pandemic-related decline may have resulted from limited *C. difficile* transmission caused by improved infection control practices in hospitals during the pandemic.

Previous studies utilizing the NIS database reported colectomy rates of 5.87 per 1,000 patients during 2001–2005, increasing to 7.72 per 1,000 patients in 2006–2010.^20^ In contrast, our study found substantially lower rates, ranging from 2.8 to 3.4 per 1,000 CDI patients between 2018 and 2022. This reduction suggests improvements in medical management strategies and potentially more selective surgical criteria. The observed decline in colectomy rates may also reflect epidemiological shifts in circulating *C. difficile* strains. A multicenter U.S. study reported that the proportion of the hypervirulent NAP1/BI/027 strain that is associated with severe and fulminant disease declined significantly from 31% in 2011–2012 to 14% in 2015–2017.^21^ This reduction in the prevalence of hypervirulent strains likely contributed to a lower incidence of fulminant CDI requiring surgical intervention. However, the modest increase of postoperative mortality observed during our study period despite declining overall CDI prevalence warrants attention. This finding contradicts expected trends and may reflect improved recognition of surgical indications among clinicians or shifts in disease severity patterns rather than treatment failure.

Postcolectomy mortality in our cohort increased from 21.0% in 2018 to 30.7% in 2022 with an average of 26.5% between 2018 and 2022. These rates align with historical data, as a previous NIS database study reported mortality rates >30% for the 2011–2015 period,^22^ and a 2020 meta-analysis of five observational studies reported postoperative mortality of 31.3% following total abdominal colectomy and 26.2% after loop ileostomy.^23^ The persistence of high mortality rates despite advances in perioperative care and critical care support is somewhat concerning. The upward trajectory during our study period suggests that patients requiring colectomy may present with greater disease severity, more complex comorbidities, or more advanced organ dysfunction than in previous years.

Our study identified peritonitis, sepsis, and coagulopathy as the strongest predictors for colectomy, which is consistent with previous investigations. A prior meta-analysis reported that peritonitis and septic shock were the most important clinical factors driving emergency colectomy and independently predicted postsurgical mortality.^10^ A 2013 NIS database study also reported that patients with CDI who developed coagulopathy had significantly higher odds of both requiring colectomy and experiencing postoperative mortality.^20^ Other factors associated with mortality following colectomy included vasopressor requirements, mechanical ventilation needs, and renal failure, all markers of multiorgan dysfunction that suggest advanced disease progression. Therefore, current clinical guidelines emphasize organ dysfunction, hemodynamic instability, and clinical deterioration as key surgical indicators.^3^

The timing of surgery is an important determinant of survival as has been suggested by previous studies, with earlier colectomies associated with decreased mortality.^7^ However, while previous research often categorized “early” surgery within a broad 7–10 day window, our multivariable analysis characterized the incremental risk: each additional day from admission to colectomy was independently associated with a 16% increase in mortality risk. That this deterioration persists independently of disease severity and comorbidities suggests a limited therapeutic window for effective intervention. The high mortality rate even with surgical intervention highlights that while early surgery improves outcomes, CDI requiring colectomy remains a life-threatening condition where timing of the surgery remains one of the few modifiable factors influencing survival.

Our findings support several clinical and operational strategies to optimize outcomes. First, patients presenting with peritonitis, coagulopathy, septic shock, or progressive organ dysfunction warrant immediate surgical consultation, even if medical therapy has just been initiated. Second, the establishment of multidisciplinary CDI response teams incorporating surgeons, intensivists, and infectious disease specialists from admission could facilitate rapid recognition of surgical candidates and expedite decision-making. Third, institutional protocols defining clear surgical triggers and timing thresholds may reduce practice variation and prevent harmful delays. The resource utilization implications are equally important. Our data suggest that delayed intervention not only worsens clinical outcomes but substantially increases healthcare costs through prolonged intensive care stays, additional organ support requirements, and management of complications.

Our study has several limitations. The observational design inherently limits causal inference, and unmeasured confounders may influence observed associations. However, our large sample size of 240,000 CDI cases representing 1.2 million patients nationally, combined with consistent results across complementary modeling approaches, strengthens confidence in our identified predictors. Administrative coding presents inherent limitations, as ICD-10 codes cannot fully capture clinical nuances such as vasopressor doses, lactate trends, or radiographic findings that influence surgical decision-making. While we employed validated tools including the Elixhauser Comorbidity Index and Patient Safety Indicators to capture clinically relevant factors, granular physiologic data remains unavailable in administrative datasets. The NIS database lacks information regarding specific medical therapies, including fidaxomicin use, bezlotoxumab administration, or fecal microbiota transplantation, which may have influenced the decision or timing of colectomy. In addition, the NIS does not include present-on-admission indicators, preventing the classification of CDI into NHSN categories (hospital-onset vs community-onset). We also cannot determine specific reasons for surgical delays, whether reflecting medical optimization attempts, diagnostic uncertainty, resource constraints, or shared decision-making processes, limiting our ability to identify modifiable delay factors. Hospital-level variation in coding practices and surgical criteria may influence apparent prevalence and outcome rates, though the national scope of our data likely mitigates single-center biases. Finally, the database structure prevents longitudinal follow-up beyond index hospitalization, potentially missing delayed mortality, readmissions, and long-term functional outcomes that would provide a more complete picture of surgical efficacy.

Our study of over 240,000 CDI hospitalizations provides further evidence that surgical timing is an important and modifiable determinant of survival in patients with severe CDI. The increase in odds of mortality associated with delayed colectomy highlights a narrow therapeutic window for effective intervention. Although the national prevalence of CDI has declined, the high mortality rate among surgical patients underscores the severity of fulminant CDI. Our findings suggest that early identification of surgical candidates and prompt intervention are important to optimize patient survival.

## Supporting information

Chen et al. supplementary material Chen et al. supplementary material

## Supplementary material

The supplementary material for this article can be found at https://doi.org/10.1017/ice.2026.10411.

## Manuscript preparation

All authors contributing to this study met the authorship criteria. No other parties were involved in the gathering of data or preparation of this manuscript.
