# Supplementary material for: Trends, predictors, and association of surgical timing with mortality among patients with Clostridioides difficile infection requiring colectomy: a cohort study
Source: Infect Control Hosp Epidemiol. 2026 Mar 5;47(5):457–69. doi: 10.1017/ice.2026.10411 (PMC13216804; doi:10.1017/ice.2026.10411)

**SUPPLEMENTARY MATERIAL**

**1A: Data Source**

The NIS is the largest publicly available all-payer inpatient healthcare database in the United States containing a 20% stratified sample of discharges from all community hospitals participating in HCUP.^1,2^ The database includes more than 7 million hospital stays annually, representing approximately 35 million hospitalizations nationally when survey weights are applied and covers more than 97 percent of the U.S. population.^1^ The NIS database uses a hospital discharge as its unit of reporting. The database includes data on patient demographics, diagnosis, and procedure coded using the International Classification of Diseases, 10th version, Clinical Modification (ICD-10-CM). The database uses complex sampling design with hospital-level clustering, stratification, and temporal sampling changes.^2^ Clinical and resource utilization data from discharge abstracts with state and hospital identifiers removed to protect confidentiality.^1^

**1B:** **Outcomes and covariates determination**

In-hospital mortality (all cause) was defined as death from any cause prior to hospital discharge. LOS represents the duration between admission and discharge dates. Total hospitalization charges included all hospital services but excluded professional fees and non-covered charges and were adjusted for inflation to 2022 dollars using the Consumer Price Index for Medical Care (CPI-M) (**Table S2**).^3^ ED admission was identified through ED revenue codes, charges, current procedural terminology (CPT) procedure codes, or admission source documentation. Discharge disposition was classified as routine home discharge versus non-home discharge (transfers to short-term hospitals, skilled nursing facilities, intermediate care facilities, other facility types, home health care services, discharge against medical advice, or unknown destinations).  Six post-operative complications following colectomy were evaluated using ICD-10-CM codes: cerebrovascular accident, pulmonary complications, cardiac complications, acute renal failure, postoperative bleeding, and urinary tract infection.

Patient-level covariates included demographics (age, sex, race/ethnicity, primary insurance, socioeconomic quartile based on median household income for patient's ZIP code) and urban-rural classification defined by metropolitan and micropolitan population estimates. Hospital characteristics included type of admission (elective vs. non-elective), admission day (weekday/weekend), transfer in and transfer out indicators, hospital census division, hospital size, hospital region, hospital location/ teaching status and hospital ownership. Colectomy day was defined as the hospital day on which the colectomy procedure was performed since admission.

Comorbidity measures were captured using the Elixhauser Comorbidity Software Refined for ICD-10-CM Diagnosis, v2025.1, limited to 20 conditions independent of present-on-admission indicators to ensure they represented pre-existing conditions rather than hospital complications. ^4,5^ CDI-related complications (electrolyte abnormalities, sepsis/septic shock, coagulopathy, peritonitis, and colonic perforation) were identified using ICD-10-CM Patient Safety Indices (PSI). **Table S1** lists the complications for CDI and colectomy, along with their corresponding ICD-10-CM code.

**1C. Patient characteristics comparison (colectomy vs non-colectomy)**

Patients who underwent colectomy were significantly younger (median age 66 vs 69 years, p < 0.001), more likely to have private insurance (24.0% vs 17.9%, p < 0.001) and more frequently treated at large (59.6% vs 50.8%, p < 0.001) and urban teaching hospitals (81.6% vs 72.5%, p < 0.001). Elective admissions were more common in the colectomy group (9.6% vs 6.6%, p = 0.007), as were transfers from other acute care hospitals (15.3% vs 8.5%, p < 0.001).

Patients who underwent colectomy had lower rates of several comorbidities, including alcohol abuse (4.1% vs 7.2%, p = 0.004), dementia (5.4% vs 10.5%, p < 0.001), diabetes (with chronic complications: 4.9% vs 7.8%, p = 0.02; and without: 21.4% vs 26.7%, p = 0.005), and hypertension (both complicated: 31.9% vs 37.8%, p = 0.004; and uncomplicated: 22.2% vs 30.7%, p < 0.001. The median comorbidity index for 30-day readmission risk was lower in patients who underwent colectomy (2 vs 4, p < 0.001).

**Supplementary References**

1. *Healthcare Cost and Utilization Project (HCUP). Overview of the Nationwide Inpatient Sample (NIS).Agency for Healthcare Research and Quality.* . 2025. <https://hcup-us.ahrq.gov/nisoverview.jsp>

2. Houchens RL RD, Elixhauser A, and Jiang J. *Nationwide Inpatient Sample Redesign Final Report*. 2014. <https://hcup-us.ahrq.gov/reports/methods/2014-04.pdf>

3. *Healthcare Cost and Utilization Project (HCUP). Using Appropriate Price Indices For Analyses Of Health Care Expenditures Or Income Across Multiple Years. Agency for Healthcare Research and Quality*. 2024. <https://meps.ahrq.gov/about_meps/Price_Index.shtml#t3a3>

4. Elixhauser A, Steiner C, Harris DR, Coffey RM. Comorbidity measures for use with administrative data. *Med Care*. Jan 1998;36(1):8-27. doi:10.1097/00005650-199801000-00004

5. *Healthcare Cost and Utilization Project (HCUP). Elixhauser Comorbidity Software*

*Rrfined For ICD-10-CM Diagnosis, v2025.1. Agency for Healthcare Research and Quality*. 2024. <https://hcup-us.ahrq.gov/toolssoftware/comorbidityicd10/CMR-User-Guide-v2025-1.pdf>

**Table S1. ICD-10-CM code for all diagnoses related to outcomes, complications and exclusion criteria**

| Category | Diagnosis | ICD-10-CM Code |
| --- | --- | --- |
| Cohort | *Clostridioides difficile* Infection | A04.71, A04.72 |
|  | Colectomy | 0DBE0ZZ, 0DBE3ZZ, 0DBE7ZZ, 0DBE8ZZ,  0DBGFZZ, 0DBLFZZ, 0DBMFZZ, 0DBNFZZ, 0DTMFZZ, 0DTE4ZZ, 0DTE0ZZ, 0DTE7ZZ, 0DTE8ZZ |
| Exclusion Criteria | Ulcerative colitis | K51. |
|  | Malignant neoplasm of colon | C18. |
|  | Acute (reversible) ischemia of large intestine | K55.03 |
|  | Acute infarction of large intestine | K55.04 |
|  | Acute (reversible) ischemia of intestine, part unspecified | K55.05 |
|  | Acute infarction of intestine, part unspecified | K55.06 |
|  | Other vascular disorders of intestine | K55.8 |
|  | Vascular disorder of intestine, unspecified | K55.9 |
|  | Melena | K92.1 |
|  | Gastrointestinal hemorrhage, unspecified | K92.2 |
| Complications of CDI | Electrolyte abnormalities | E87 |
|  | Sepsis/septic shock | A40, A41 |
|  | Coagulopathy | D65-D69 |
|  | Peritonitis | K65 |
|  | Colonic perforation | K63.1 |
| Post-operative colectomy complications | Cerebrovascular accident | I97.82 |
|  | Pulmonary complications | J95.1, J95.2, J95.89, T81.718 |
|  | Cardiac complications | I97.89, I21, T81.1 |
|  | Acute renal failure | N17.9 |
|  | Postoperative bleeding | K91.840 |
|  | Urinary tract infection | N39.0 |

**Table S2. Consumer Price Index - Medical Care (CPI-M)**

| Year | CPI-M |
| --- | --- |
| 2018 | 484.707 |
| 2019 | 498.413 |
| 2020 | 518.876 |
| 2021 | 525.276 |
| 2022 | 546.554 |

Figure S1. Receiver Operating Characteristic (ROC) Curves Comparing Least Absolute Shrinkage and Selection Operator (LASSO)and Multivariate Logistic Regression Models for Predicting Colectomy in Discharges with CDI


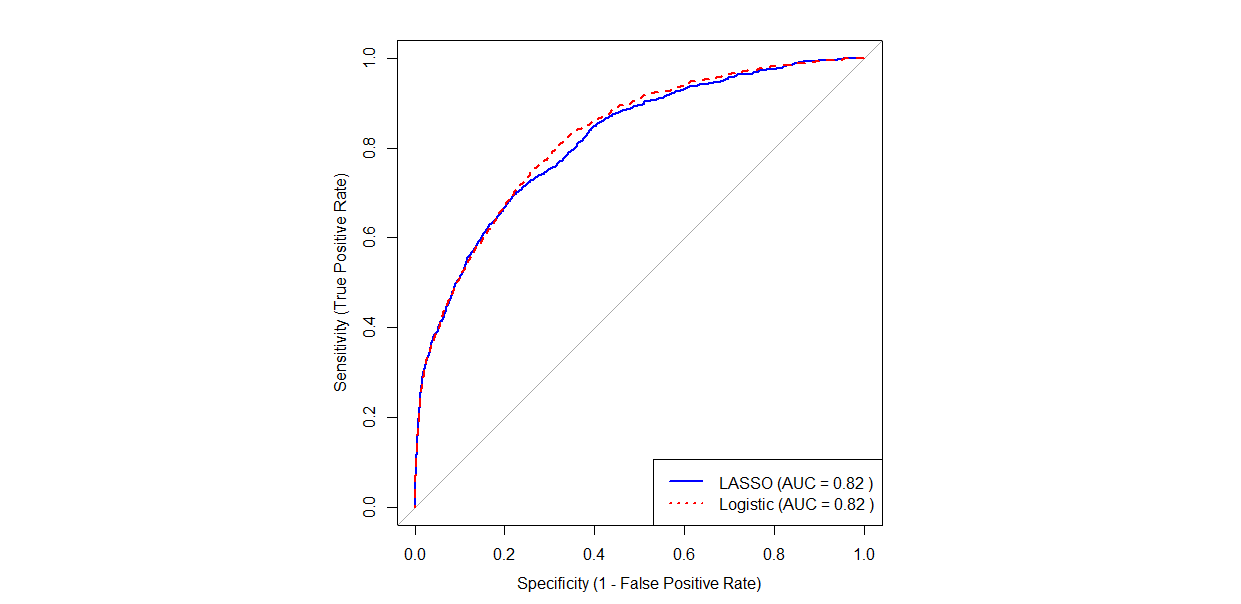


Figure S2. Receiver Operating Characteristic (ROC) Curves Comparing Least Absolute Shrinkage and Selection Operator (LASSO) and Multivariate Logistic Regression Models for Predicting Mortality in Discharges with CDI and Colectomy


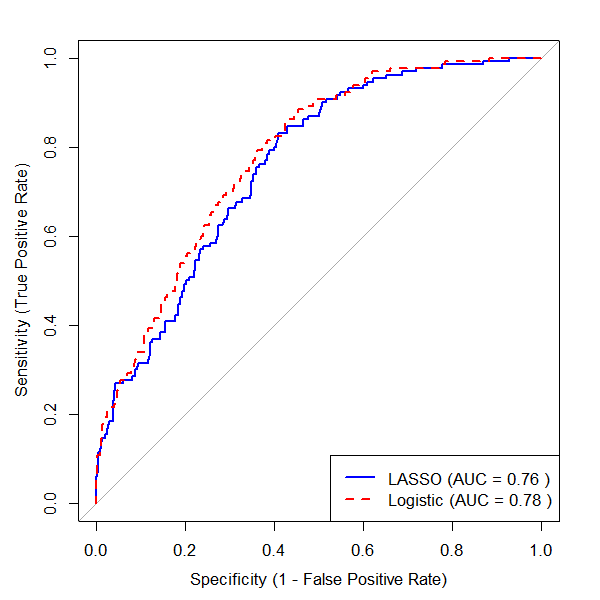

Supplement: Chen et al. supplementary material [file S0899823X26104115sup001.docx]
